# Lipolytic and Lipophagic Effects of *Pinellia ternata* Pharmacopuncture on Localized Adiposity

**DOI:** 10.1155/2021/7347639

**Published:** 2021-01-06

**Authors:** Haesu Lee, Mi Hye Kim, Seong Chul Jin, Jae Min Han, Jun Hyuk Park, Woong Mo Yang

**Affiliations:** Department of Convergence Korean Medical Science, College of Korean Medicine, Graduate School, Kyung Hee University, 26 Kyungheedae-ro, Dongdaemun-gu, Seoul 02447, Republic of Korea

## Abstract

Localized adiposity is not only a common aesthetic issue but also a health risk factor. Pharmacopuncture can be a therapeutic option for the imbalance of regional fat distribution. The tuber of *Pinellia ternata* has been prescribed as antitussive and expectorant as a traditional Korean medicine. This study investigated the effects of pharmacopuncture with *P. ternata* water extract (PT) on localized adiposity. Male C57BL/6J mice were fed on a high-fat diet (HFD) for 6 weeks. 100 *μ*L of 10 mg/mL of PT was injected into the left-side inguinal fat pad, while saline was injected into the right-side inguinal fat pad as self-control. Treatments were performed 3 times per week for 4 weeks. The inguinal fat weight was analyzed by dual-energy X-ray absorptiometry. PT pharmacopuncture significantly decreased the weight of the inguinal fat pad. The adipocyte size was reduced with increases of lipolytic enzymes and lipophagy-related factors by PT pharmacopuncture. There was marked inhibition of lipid accumulation content in 3T3-L1 adipocytes by PT treatment. The expressions of adipose triglyceride lipase (ATGL), hormone-sensitive lipase (HSL), autophagy-related gene (ATG) 5, ATG7, and LC3 were markedly increased by PT treatments *in vivo* and *in vitro*. This study suggests that pharmacopuncture of *Pinellia ternata* has ameliorative effects on adiposity by lipid catabolic effects via activating both lipolysis and lipophagy in a localized region.

## 1. Introduction

Abnormal and excessive lipid storage in adipose tissues is a main characteristic of obesity, defined as a body mass index (BMI) greater than or equal to 30 kg/m^2^ [1]. Triglyceride (TG) is a major constituent of lipid droplets (LDs) in white adipose tissues [2]. The regional distribution of TG deposition in visceral, abdominal, and subcutaneous fat, regarded as localized adiposity, is closely associated with the high risk of obesity rather than BMI [3]. For that reason, hydrolysis of TG is a vital therapeutic target not only to reduce physical inconvenience but also to improve secondary metabolic diseases such as hypertension, diabetes mellitus, cardiac disorders, and cancer in obese conditions [4].

The aesthetic treatment for obesity is commonly performed in subcutaneous adipose tissue of the abdomen [5]. Liposuction, extraction of fat tissue by suction, is one of the most frequently performed cosmetic procedures in North America [6]. While liposuction can remove the subcutaneous fat effectively with tumescent technique and small diameter cannula, complications can vary from cannula injury and infection to embolism and contour deformity and even death [7]. Phosphatidylcholine (PC) and/or deoxycholate (DC) injections have been widely used as a nonsurgical option for the correction of localized fat [8]. PC and DC injections could reduce local fat deposits by the lipolytic effects [9]. However, severe adverse effects of injection lipolysis with PC and DC have been reported such as tissue necrosis and fibrosis [10]. Pharmacopuncture, injection of natural herbs at the acupuncture point, is a new acupuncture technique in traditional Korean medicine and is able to be an alternative to liposuction and PC-DC injection [11]. Several natural herbs including *Ephedra sinica* Staph., *Atractylodes ovata* (Thunb.) DC., *Panax quinquefolius* L., and *Eriobotrya japonica* (Thunb.) Lindl. have been studied in pharmacopuncture for obesity [12].

The tuber of *Pinellia ternata* (Thunb.) Breitenb. (Araceae) has been prescribed as an antitussive and expectorant agent for throat pain, asthma, and chronic obstructive pulmonary disease in traditional Korean medicine [13]. Currently, *P. ternata* has been demonstrated to have inhibitory effects on vomiting, infection, inflammation, and cancer [14–17]. Kim et al. have reported that oral administered *P. ternata* has an antiobesity effect on Zucker rats by converting from fat depots into brown adipose tissue which is thermogenically active [18]. This study can be supported by theories of traditional Korean medicine in which *P. ternata* is known to be an efficient herb for dispelling “phlegm dampness” that is the leading cause of obesity [19]. Interestingly, a herbal formula containing *P. ternata*, Weikang Keli, has been shown to induce autophagy on the gastric cancer cell, which is confirmed by detecting biomarkers for autophagic cells including microtubule-associated protein 1 light chain 3 (LC3) [20].

However, the effect of *P. ternata* pharmacopuncture on localized obesity has not been researched. In the present study, we investigated the effect of pharmacopuncture with *P. ternata* water extract (PT) on inguinal fat pad of obesity-induced mice by analyzing local fat loss, adipose tissue histology, and the activities of adipose triglyceride lipase (ATGL), hormone-sensitive lipase (HSL), LC3, autophagy-related gene (ATG) 5, and ATG7. In addition, the effects of PT on 3T3-L1 adipocyte-like cells were demonstrated by measuring the levels of lipid content, ATGL, HSL, LC3, ATG5, and ATG7 with and without the addition of lipophagy inhibitor.

## 2. Materials and Methods

### 2.1. Preparation of Samples

PT processed with ginger juice and alum known as Kangbanha in Korea was purchased from Dong-Yang Herb Inc. (Seoul, Korea). To avoid the toxicity of raw PT, herb manufacturer provides PT processed with ginger juice and alum in Korea. 10 g of PT was extracted with 150 mL of distilled water at 25°C for 2 h. The extract was concentrated under decompression filtration. It was powdered by freeze dryer (yield: 5.76%). The sample was stored at −20°C until use.

### 2.2. Animal Treatment

**Figure 1:**
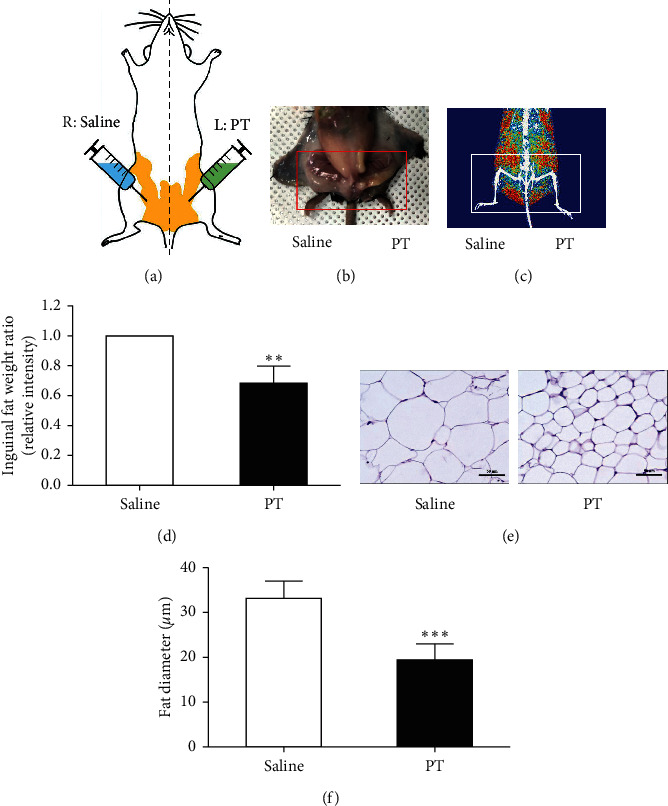
Schematic diagram of sample treatment (a). Morphological changes of the inguinal fat pad (b) and representative picture from DXA (c). Red dots indicate the deposition of fat tissues visualized by the DXA program. The ratio of inguinal fat pad weight (d). The inguinal fat was weighed by DXA. Representative picture of histological changes of fat tissues (e) and quantified fat diameter of adipocyte (f). Localized adiposity and size of adipocyte were evaluated stained with H&E. Results are presented as mean ± standard deviation. ^*∗∗*^*p* < 0.01 and ^*∗∗∗*^*p* < 0.001 vs. saline-treated right-side fat as self-control. PT, *Pinellia ternata*.

Five-week-old male C57BL/6J mice were purchased from Raonbio Inc. (Yongin, Korea). Eight mice were housed under 22 ± 2°C temperature and 50 ± 5% humidity-controlled facility. After 1 week of housing, 10 mice were fed a high-fat diet (HFD) containing 60% fat to induce obesity. Following 6 weeks of HFD feeding, 10 mg/mL of PT was injected to left inguinal fat pad (PT), while saline was injected into the right inguinal fat pad (saline) for self-control (Figure 1(a)). The injected volume of saline and PT sample was 100 *µ*L per mice, respectively. The treatment was performed 3 times per week for 4 weeks. Body weight was measured once a week through whole animal experiments. All animal procedures were approved by the Committee on Care and Use of Laboratory Animals of the Kyung Hee Univ. (KHUASP (SE)-18-070).

### 2.3. Measurement of Inguinal Fat Weight by Dual-Energy X-Ray Absorptiometry

At the end of the animal experiment, the mice were anesthetized. Dual-energy X-ray absorptiometry (DXA; Medikors, Seongnam, Korea) was used to analyze the fat weight of the inguinal region. The determined region was from knee to tail based on a line of the ventral spine. The fat decomposition was indicated by a red dot.

### 2.4. Histology

Both sides of inguinal fat pads were dissected, respectively. The specimens were fixed with 10% neutralized formalin for 24 h. Then, fat tissues were dehydrated with gradient ethanol and xylene and embedded in paraffin. The prepared paraffin blocks were cut into 5 *μ*m using a microtome (HM355S, Thermo Scientific, UK). Each tissue slide was stained with hematoxylin and eosin to confirm the structure and diameter of the adipocyte in the inguinal fat pad. Histological changes were observed under an optical microscope (Leica DM 500; Leica, Wetzlar, Germany). Five slides of each mouse were randomly selected, and the diameter of adipocyte was measured using an automated analysis program ImageJ (National Institutes of Health, Bethesda, MD, USA).

### 2.5. Measurement of Serum Toxicity

Blood samples were collected by cardiac puncture and centrifuged at 17000 rpm for 30 min. The supernatant was separated and used for estimation of BUN, Creatinine, AST, and ALT by enzyme-linked immunosorbent assay (ELISA) according to the manufacturer's instruction.

### 2.6. Adipocytes Differentiation by Oil Red O Staining

3T3-L1 murine preadipocytes were purchased from the American Type Culture Collection (Rockville, MD, USA). The cells were grown in Dulbecco's modified eagle medium (DMEM) with 10% bovine serum (BS) at 37°C with 5% CO_2_. After cells confluence on plates (Day 0), differentiation media (MDI) containing 10% fetal bovine serum (FBS), 1% penicillin-streptomycin (P/S), 0.5 mM 3-isobutyl-1-methylxanthine (IBMX), 1 *μ*M dexamethasone, and 5 *μ*g/mL insulin in DMEM were incubated into cells until Day 3. The differentiation media were then changed to second differentiation media containing 10% FBS, 1% P/S, and 5 *μ*g/mL insulin in DMEM for another 2 days. On Day 5, fresh second differentiation media were incubated again for additional 2 days. On Day 7, 3T3-L1 preadipocytes were differentiated into mature adipocytes. 1, 10, and 100 *μ*g/mL of PT dissolved in distilled water were treated to MDI-induced mature adipocytes for 24 h. In addition, 0.2 mM 3-methyladenine (3-MA), a lipophagic inhibitor, with 1, 10, and 100 *μ*g/mL of PT was incubated into 3T3-L1 differentiated cells for 24 h. Finally, the cells were fixed with 5% neutralized formalin to measure the levels of lipid accumulation using the Oil Red O (ORO) staining method on Day 8. After incubation with 0.5% Triton X-100 for 10 min, the cells were rinsed with 60% of isopropanol for 5 min and completely dried. 60% filtered ORO working solution in distilled water was added to 3T3-L1 cells to stain LD at room temperature for 2 h. To visualize cellular LD, ORO-stained cells were observed by a microscope. In addition, relative lipid accumulation content compared to the control was quantified by an additional extraction procedure. 100% isopropanol was added to each well. The supernatant was transferred into a 96-well plate. The absorbance was measured at 500 nm using an ELISA microplate reader (BioTek, PA, USA).

### 2.7. Western Blot Analysis

Inguinal fat tissue was homogenized with tissue protein extraction buffer (Thermo Scientific, Rockford, USA) with protease inhibitor cocktails (Roche, Mannheim, Germany). The lysate was centrifuged at 17000 rpm for 15 min and the supernatant was collected. The protein concentrations were calculated by Bradford assay solution (Bio-Rad, CA, USA). 10 *μ*g protein was separated on 10% SDS-PAGE and then transferred onto polyvinylidene fluoride (PVDF) membranes (Bio-Rad, CA, USA). The membranes were probed with the primary antibodies specific for *β*-actin, ATGL, HSL, LC3, ATG5, and ATG7 (Cell Signaling Technology, Danvers, MA, USA) at 4°C overnight. Secondary antibodies were incubated with horseradish peroxidase-conjugated anti-mouse or anti-rabbit (Santa Cruz Biotechnology, CA, USA) for 1 h at room temperature. The protein bands were detected with chem-luminescence reagents (AbClon, Seoul, Korea) by chemi-doc (Davinch-K, Seoul, Korea). *β*-Actin, as a loading control, was used to normalize the levels of proteins.

### 2.8. Statistical Analysis

Significance was determined by one-way analysis of variance (ANOVA) and Tukey's multiple comparison tests. In all analyses, *p* < 0.05 was taken to indicate statistical significance.

## 3. Results

### 3.1. Effects of PT Pharmacopuncture on the Inguinal Fat Weight in Obese Mice

The weight of the left inguinal fat tissues injected with PT pharmacopuncture (PT) was markedly reduced compared to that of the right inguinal fat tissues injected with saline (saline) (Figures 1(b) and 1(c)). Specifically, the fat pad administered with physiological saline was measured to be 149.2 ± 7.5 mg, while the fat pad treated with PT weighed 107.5 ± 16.1 mg. When the saline treatment group was converted to 1, the relative value of PT-injected fat pad weight was estimated to 0.73 ± 0.14, indicating that PT pharmacopuncture decreased the weight of inguinal fat pad by about 27% compared to the saline control (Figure 1(d)).

### 3.2. Effects of PT Pharmacopuncture on Histological Changes of Inguinal Fat Tissues in Obese Mice

Hematoxylin and eosin staining was performed to confirm the histological change of localized adipose tissues and measure the diameter of adipocyte diameters. Injection of PT pharmacopuncture dramatically inhibited the size of adipocyte in inguinal fat pad tissues of HFD-fed obese mice (Figure 1(e)). HFD feeding induced 132.9 ± 3.5 *μ*m of adipocyte diameter in the inguinal fat pad. The diameter of the adipocyte in adipose tissues was 79.5 ± 7.2 *μ*m by PT injection. When the diameter of adipocyte in the saline-treated fat pad was converted to 1, the relative value of adipocyte diameter in PT-treated fat pad was estimated to 0.6 ± 0.06, indicating that PT pharmacopuncture significantly decreased the adipocyte diameter of the inguinal fat pad by about 40% compared to the saline control (Figure 1(f)).

### 3.3. Effects of PT Pharmacopuncture on the Expressions of Lipolytic Enzymes in Inguinal Fat Tissues in Obese Mice

**Figure 2:**
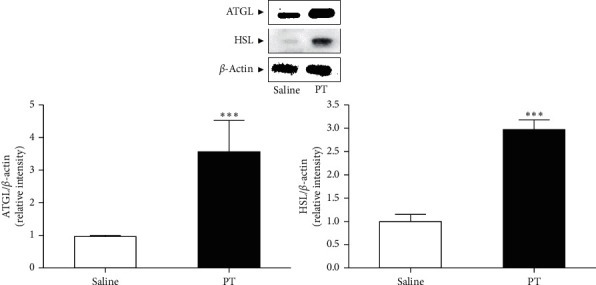
The expressions of lipolytic enzymes in inguinal fat tissues of HFD-induced obese mice. The levels of ATGL and HSL protein (*n* = 10) were visualized by Western blot analysis. Results are presented as mean ± standard deviation. ^*∗∗∗*^*p* < 0.01 vs. saline-treated right-side fat as self-control. The experiments were carried out in triplicate measurements. PT, *Pinellia ternata*; ATGL, adipose triglyceride lipase; HSL, hormone-sensitive lipase.

The expression of lipolysis-related factors such as ATGL and HSL was determined in fat tissues of saline and PT group, respectively. PT injection significantly increased the ATGL expression by about 3.4 times compared to saline. In addition, there was a 2.9-fold increase of HSL protein expression in the PT-injected right side of the inguinal fat pad compared to the self-control saline side (Figure 2).

### 3.4. Effects of PT Pharmacopuncture on Expressions of Lipophagy-Related Factors in Inguinal Fat Tissues in Obese Mice

**Figure 3:**
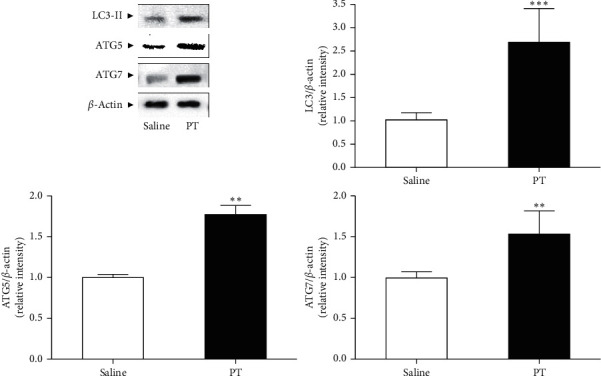
The expressions of lipophagic factors in inguinal fat tissues of HFD-induced obese mice. The levels of LC3-II, ATG5, and ATG7 protein were visualized by Western blot analysis. Results are presented as mean ± standard deviation. ^*∗∗*^*p* < 0.01 and ^*∗∗∗*^*p* < 0.01 vs. saline-treated right-side fat as self-control. The experiments were carried out in triplicate measurements. PT, *Pinellia ternata*; LC3-II, microtubule-associated protein 1A/1B-light chain 3-II; ATG5, autophagy-related gene 5; ATG7, autophagy-related gene 7.

The expression of lipophagy-related factors such as LC3, ATG5, and ATG7 was determined in fat tissues of saline and PT group, respectively. PT injection significantly increased the LC3 expression by about 2.6 times compared to saline-injected fat tissue. ATG5 and ATG7 expressions in HFD-induced fat tissues were significantly increased by treatment with PT pharmacopuncture by about 1.7 times and 1.5 times (Figure 3).

### 3.5. Effects of PT on Lipid Accumulation in 3T3-L1 Adipocytes

**Figure 4:**
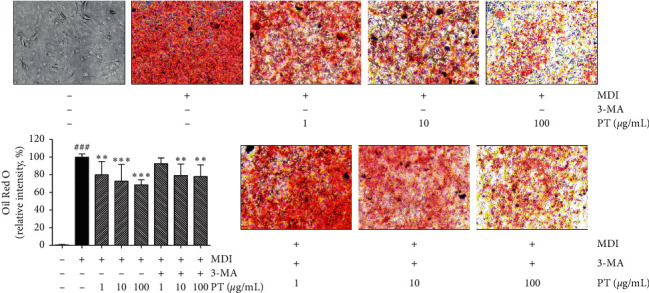
Intracellular lipid accumulation content in differentiated 3T3-L1 adipocytes. Intracellular lipid accumulation was conducted by ORO staining. Results are presented as mean ± standard deviation. ^###^*p* < 0.001 vs. undifferentiated cells; ^*∗*^*p* < 0.05, ^*∗∗*^*p* < 0.01, and ^*∗∗∗*^*p* < 0.01 vs. MDI-induced differentiated cells. The experiments were carried out in triplicate measurements. PT, *Pinellia ternata*; ORO, Oil Red O; 3-MA; 3-methyladenine.

Intracellular lipid accumulation was observed by ORO staining in differentiated 3T3-L1 cells. Differentiated cells induced by MDI and additional insulin incubation showed red-stained hypertrophic adipocytes when compared to the nontreated control. There was abundance in the round-shaped intracellular LDs in differentiated 3T3-L1 cells. 1, 10, and 100 *μ*g/mL of PT treatment dose-dependently inhibited the TG accumulation content by about 19.92%, 27.3%, and 31.32% compared to differentiated adipocytes. In the presence of 3-MA, PT treatment lowered the intracellular lipid content in a dose-dependent manner by about 7.42%, 20.8%, and 22.02% in 1 *μ*g/mL of PT + 3-MA, 10 *μ*g/mL of PT  + 3-MA, and 100 *μ*g/mL of PT + 3-MA, respectively. The LD was significantly decreased even if PT extract was cotreated with 3-MA, an inhibitor of autophagy. However, the inhibition rates of lipid accumulation in cells cotreated with PT and 3-MA were lower than cells treated with only PT (Figure 4).

### 3.6. Effects of PT on Expressions of Lipolytic Enzymes in Differentiated 3T3-L1 Adipocytes

**Figure 5:**
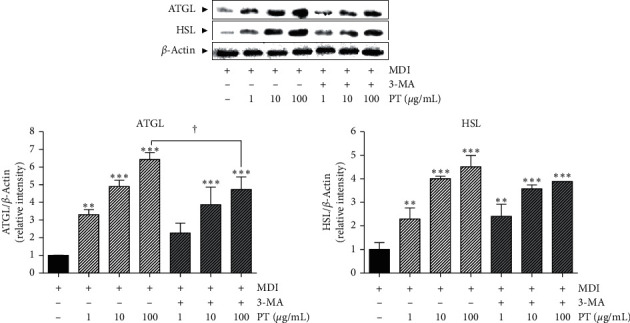
The expressions of lipolytic enzymes in differentiated 3T3-L1 adipocytes. The levels of ATGL and HSL protein were visualized by Western blot analysis. Results are presented as mean ± standard deviation. ^*∗*^*p* < 0.05, ^*∗∗*^*p* < 0.01, and ^*∗∗∗*^*p* < 0.01 vs. MDI group. PT, *Pinellia ternata*; ATGL, adipose triglyceride lipase; HSL, hormone-sensitive lipase.

PT treatment in differentiated 3T3-L1 cells showed a dose-dependent effect on increases of ATGL and HSL expression. Compared with MDI-induced differentiated cells, the increased rates of ATGL by treatment with 1, 10, and 100 *μ*g/mL of PT were 231%, 390.7%, and 543%. Also, cells treated with PT in the presence of 3-MA showed a similar increase % by cells treated with only PT (126.7%, 287%, and 373.4% in 1 *μ*g/mL of PT + 3-MA, 10 *μ*g/mL of PT + 3-MA, and 100 *μ*g/mL of PT + 3-MA, respectively). In addition, PT 1, 10, and 100 *μ*g/mL treatment significantly increased the HSL expressions by about 129.7%, 300.5%, and 352% compared to MDI-stimulated differentiated cells. There was no difference in the expression of HSL in differentiated adipocytes between only PT-treated cells and PT and 3-MA-cotreated cells (Figure 5).

### 3.7. Effects of PT on Expressions of Lipophagy-Related Factors in Differentiated 3T3-L1 Adipocytes

**Figure 6:**
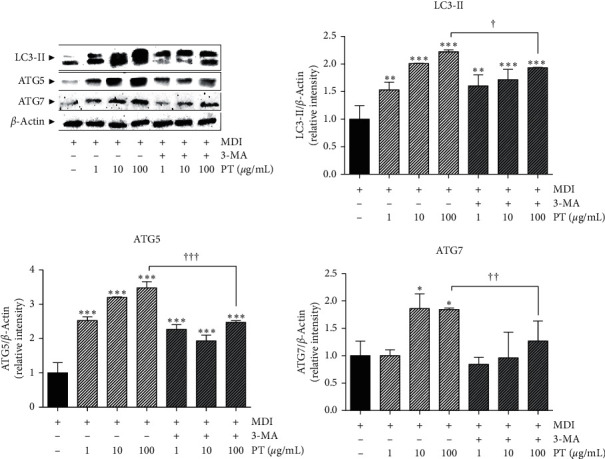
The expressions of lipophagic factors in differentiated 3T3-L1 adipocytes. The levels of LC3, ATG5, and ATG7 protein were visualized by Western blot analysis. Results are presented as mean ± standard deviation. ^*∗*^*p* < 0.05, ^*∗∗*^*p* < 0.01, and ^*∗∗∗*^*p* < 0.01 vs. MDI-induced differentiated cells. PT, *Pinellia ternata*; LC3, microtubule-associated protein 1 A/1B-light chain 3; ATG5, autophagy-related gene 5; ATG7, autophagy-related gene 7.

The expression of LC3 following induction of differentiation was found to be significantly increased by PT treatment in 3T3-L1 cells. In particular, treatment with 100 *μ*g/mL of PT markedly raised the 1.22-fold LC3 expression. Cotreatment of PT with 3-MA also increased the expression of LC3; however, the increased rates at all concentrations of PT were less than only 100 *μ*g/mL of PT-treated cells. Compared to MDI-induced differentiated cells, the expressions of ATG5 and ATG7 were significantly increased by 10 and 100 *μ*g/mL of PT treatment. In cells treated with 100 *μ*g/mL of PT with 3-MA, a slight change of those factors was shown (Figure 6).

### 3.8. Effects of PT Pharmacopuncture on Body Weight in Obese Mice

Through the whole animal experiment, body weight was confirmed every 1 week to check the condition of mice. Because mice were fed with HFD, body weight was gradually increased during the induction period of obesity. Following the HFD feeding, the body weight of all mice was consistently increased during periods of the PT pharmacopuncture treatments, indicating that PT pharmacopuncture did not affect the body weight (Table 1).

### 3.9. Effects of PT Pharmacopuncture on Serum Hepatoxicity and Nephrotoxicity in Obese Mice

The levels of injury marker of liver and kidney were investigated to assess the potential toxic effect of PT. The serum levels of AST and ALT were 174.60 ± 38.49 U/L and 28.40 ± 4.72 U/L in PT-treated mice. Serum AST and ALT levels were within the normal range without hepatoxicity by PT treatment. Also, the levels of BUN and creatinine as nephrotoxic markers in PT-treated mice were 29.00 ± 2.92 mg/dL and 0.29 ± 0.03 mg/dL, indicating that there was no toxicity such as hepatoxicity and nephrotoxicity by PT treatment to HFD-induced obese mice (Table 2).

## 4. Discussion

Localized adiposity is not only a common aesthetic issue but also a health risk factor [21]. Due to the imbalance of regional fat distribution, local administrations including pharmacopuncture may be needed to reduce the localized expansion of fat mass [22]. Fat accumulation primarily relies on the balance between lipolysis and lipogenesis in adipose tissue [23]. Mature adipocytes are responsible for this dynamic metabolism of LDs with a unique property in cell size alteration [24]. During the fat breakdown, the size of adipocytes gets smaller as LDs shrink. However, fat synthesis is accompanied by increases in size as well as the number of adipocytes [25]. In this study, PT pharmacopuncture was injected into the left inguinal fat pad in HFD-induced obese mice with a saline injection into the right side as self-control. PT pharmacopuncture significantly decreased the weight of inguinal fat pad and the size of adipocytes in adiposity induced mice, which means that PT pharmacopuncture seems to have effects on fat degradation.

Lipolysis is a hydrolytic process of TG in LDs to triacylglycerol-associated free fatty acids (FFAs) and glycerol for energy production in regular sequence [26]. Lipolytic activity in obesity is characterized by an overall increase with highly maintained basal lipolysis and a decrease in catecholamine-stimulated lipolysis by a defect in insulin-mediated suppression [27]. It is well established that the two major cytosolic enzymes, ATGL and HSL, mediate the hydrolysis of TG. In the present study, expressions of ATGL and HSL were markedly increased by PT pharmacopuncture indicating that PT has a lipolytic effect by local treatment.

In addition to lipolysis, lipophagy is another pathway to fat breakdown and TG utilization [28]. Since defective lipophagy is thought to have a role in the pathology of obesity with lipid breakdown, insulin sensitivity, and food intake, therefore, regulating lipophagy can be a therapeutic target for adiposity and obesity [29]. In the process of lipophagy, ATGs regulate fundamental procedures of lipophagy such as autophagosome formation, transportation to the lysosome, and the fusion of the autophagosome with lysosome [30]. A pivotal component of autophagosome structure, LC3-II is formed by ubiquitin-like conjugation of LC3 and phosphatidylethanolamine, called LC3 lipidation, with the enzymic activity of ATG7 [31]. The conjugation of ATG12 with ATG5, also activated by ATG7, forms a multimeric complex with ATG16L, which contributes to the LC3 lipidation and the elongation of autophagosome membrane [32, 33]. LDs are encapsulated by an autophagosome and degraded by acidic enzymes of lysosomal organelle which is fused with the autophagosome subsequently [34]. The expressions of LC3-II and two essential ATG5 and ATG7 were significantly increased by PT treatment. The expressions of ATGs and LC3-II were decreased by PT in the presence of 3-MA compared to PT solo treatment in differentiated 3T3-L1 adipocytes, which is the first evidence that PT treatment may degrade TG by upregulating the lipophagy.

Taken together, PT treatment showed both lipolytic and lipophagic effects on localized adiposity in obese mice. We analyzed the degradative effects of PT on LDs with and without lipophagic activity in vitro. TG accumulation in the differentiation of 3T3-L1 adipocytes was significantly decreased by PT treatment. The expressions of lipolytic enzymes, including ATGL and HSL, and lipophagic proteins, including ATG5, ATG7, and LC3-II, were increased by PT significantly and dose-dependently in vitro, which means that PT has lipid degradative effects by activating both lipolysis and lipophagy.

## 5. Conclusion

PT pharmacopuncture showed the catabolic effects of localized adiposity by activating both lipolysis and lipophagy. These findings suggest that *P. ternata* pharmacopuncture would be an alternative treatment for reducing localized fat in obesity.

## Figures and Tables

**Table 1:** Body weight as time course in HFD-induced obese mice.

Weeks after treatment	Body weight (g)
−6	16.72 ± 0.35
−5	18.63 ± 0.42
−4	20.3 ± 0.75
−3	21.15 ± 0.82
−2	22.38 ± 1.63
−1	23.78 ± 2.08
0	24.27 ± 1.49
1	24.35 ± 1.86
2	26.07 ± 2.50
3	27.20 ± 3.04
4	27.08 ± 3.29

Body weight of mice was measured every 1 week. Results are presented as mean ± standard deviation. PT, *Pinellia ternata*.

**Table 2:** Serum AST, ALP, BUN, and creatinine levels in HFD-induced obese mice.

Analyte	Value in PT-treated mice	Normal range
AST (GOT) (U/L)	174.60 ± 38.49	54∼298 U/L
ALT (GPT) (U/L)	28.40 ± 4.72	20∼90 U/L
Creatinine (mg/dL)	0.29 ± 0.03	<0.5 mg/dL
BUN (mg/dL)	29.00 ± 2.92	16∼36 mg/dL

Results are presented as mean ± standard deviation. PT, *Pinellia ternata*. All experiments were approved by the Committee on Care and Use of Laboratory.

## Data Availability

The data used to support the findings of this study are available from the corresponding author upon reasonable request.

## Abbreviations

3-MA: 3-Methyladenine
ATG5: Autophagy-related gene 5
ATG7: Autophagy-related gene 7
ATGL: Adipose triglyceride lipase
BMI: Body mass index
DC: Deoxycholate
HSL: Hormone-sensitive lipase
LC3: The light chain protein 3
LD: Lipid droplets
ORO: Oil Red O
PC: Phosphatidylcholine
PT: *Pinellia ternate*
TG: Triglyceride.
